# Mental Health Outcomes Among Female Ukrainian Refugees in Germany—A Mixed Method Approach Exploring Resources and Stressors

**DOI:** 10.3390/healthcare13030259

**Published:** 2025-01-28

**Authors:** Adekunle Adedeji, Stella Kaltenbach, Franka Metzner, Viktoriia Kovach, Stefan Rudschinat, Isabel Marin Arrizabalaga, Johanna Buchcik

**Affiliations:** 1 Department of Social Work, Hamburg University of Applied Sciences, 20099 Hamburg, Germany; 2 Department of Health Sciences, Hamburg University of Applied Sciences, 21033 Hamburg, Germany; stella.kaltenbach@haw-hamburg.de (S.K.); viktoriia.kovach@haw-hamburg.de (V.K.); johanna.buchcik@haw-hamburg.de (J.B.); 3 Bremen International Graduate School of Social Sciences, Constructor University, 28759 Bremen, Germany; 4 Department of Medical Psychology, University Medical Center Hamburg-Eppendorf, 20246 Hamburg, Germany; fmetzner@uke.de; 5 Johann-Daniel-Lawaetz-Stiftung, 22763 Hamburg, Germany; rudschinat@lawaetz.de; 6 Mit Migranten für Migranten (MiMi), 22765 Hamburg, Germany; info@mimi-hamburg.de

**Keywords:** female Ukrainian refugees, mental health outcomes, psychological well-being, mixed methods approach, stressors, language barriers, acculturation stress, anxiety and depression, isolation and disconnection, coping resources

## Abstract

Background: Mental health outcomes among female refugees are complex and multifaceted. This study examines the mental health outcomes and coping resources of female Ukrainian refugees in Germany using a mixed methods approach with qualitative focus groups and quantitative assessments. Methods: This study employs a mixed methods approach, combining qualitative and quantitative methodologies. Three focus group discussions with fifteen participants were conducted in Hamburg in April 2023. A stepwise qualitative data analysis was done using a deductive coding technique. The quantitative analysis focused on descriptive statistics to summarize the data and provide an overview of participants’ mental health and well-being. Results: Our findings show that 43% of participants reported anxiety symptoms, and 21% showed signs of depression—many experienced isolation and disconnection and were struggling with cultural adaptation and the emotional toll of displacement. Despite challenges, participants utilized various coping strategies, such as staying active, volunteering, and seeking community support. However, significant barriers to accessing mental health services, especially for children, were identified. Conclusions: The study underscores the need for tailored interventions, including language support, accessible mental health resources, and community engagement, to foster resilience and well-being. It highlights the importance of comprehensive support systems for refugee populations in host countries, such as active lifestyles, social support, volunteer work, and successful integration.

## 1. Introduction

The humanitarian crisis resulting from the war in Ukraine has catalyzed a significant influx of refugees seeking safety and stability in different European countries, including Germany. Unlike many refugee crises where the demographic profile may vary, the conflict in Ukraine has resulted in a predominantly female and children refugee population due to limitations on male Ukrainians seeking asylum abroad [1]. This demographic shift presents distinct challenges, particularly concerning the mental health and well-being of women and children who often bear the brunt of displacement and conflict-related trauma [2,3].

Mental health outcomes among female refugees are complex and multifaceted. Research indicates a high prevalence of psychological distress, encompassing symptoms of depression, anxiety, and post-traumatic stress disorder (PTSD) [2,4]. Furthermore, female refugees may be more prone to various forms of violence linked with their displacement, loss of loved ones, and uncertainty about the future [4]. Such experiences have been highlighted as significant contributors to the psychological burden carried by these refugees even after arriving in places of safety. Furthermore, acculturation stress, language barriers, and social isolation in the destination country may further exacerbate mental health challenges, highlighting the need for tailored interventions that address the unique needs of this population [5,6]. Examining the mental health outcomes of female Ukrainian refugees in Germany, therefore, provides critical information due to the unique challenges and vulnerabilities they face during forced migration. Women often experience distinct stressors, including the disruption of family structures, heightened caregiving responsibilities, and exposure to trauma, which can significantly impact their psychological well-being. At the same time, they may draw on specific resources, such as social networks and resilience, to navigate these challenges [5,6]. Exploring these stressors and resources is essential for understanding the complex interplay of factors affecting their mental health. This knowledge is crucial for developing targeted interventions and policies that address their specific needs, promote resilience, and enhance their well-being during resettlement.

Previous studies on the mental well-being of Ukrainian refugees, employing quantitative analysis, indicate a significant occurrence of mental health issues as evidenced by overall psychological distress, depressive tendencies, and anxiety within the Ukrainian refugee population after arrival in Germany [7]. Buchcik et al. (2023) found that over 60% of the participants (*N* = 304) expressed notable psychological distress, citing several reasons, such as sleep disturbances due to worry, persistent feelings of pressure, and a decline in confidence or self-esteem, among other factors. Similarly, close to half of the participants exhibited signs of depression, while more than half reported experiencing anxiety symptoms characterized by nervousness, tension, and difficulties attributed to the war in their home country [7]. While these results provided a glimpse into the mental health outcomes, the quantitative approach prevents a deeper understanding of the mechanisms of how the various factors may affect the overall mental health outcome for female Ukrainian refugees.

The Ukrainian refugees are unique due to the sociopolitical dynamics of the Russian invasion of Ukraine [8]. Recent findings research findings suggest that Ukraine’s complex history may have profound implications for female Ukrainian refugees, influencing their integration prospects and access to support services in host countries in several ways [9,10]. Furthermore, political instability from conflict can lead to uncertainty and insecurity, disrupting stability and worsening psychological distress [11]. Additionally, the political dimensions of the conflict may influence the reception and treatment of Ukrainian refugees across different countries, with varying levels of sympathy or hostility towards their plight [12]. As a host country for different refugee groups, Germany has significantly provided sanctuary and support to displaced individuals worldwide [13]. The positive public reception towards Ukrainian refugees in Germany reflects a widespread sense of empathy and solidarity with their plight. Many Germans have shown a willingness to extend a warm welcome to Ukrainian refugees, offering assistance with housing, language learning, and integration into local communities [14]. However, while many people in Germany have shown compassion, it is also important to acknowledge that some Ukrainian refugees still encounter instances of racism or discrimination, which may affect the experiences of refugees as they navigate their new lives in Germany. This dual reality emphasizes the challenges and resources that significantly impact refugees’ well-being and integration [15].

Despite this warm welcome to Ukrainian refugees, poor mental health outcomes remain evident, arguably underscoring the profound impact of the war and displacement on this population [7]. One significant factor contributing to these challenges relates to the stressors and challenges associated with their integration [7,11]. For example, language barriers may hinder communication and access to essential services, exacerbating feelings of isolation and alienation. Additionally, the bureaucratic hurdles in navigating asylum procedures and securing employment or housing in Germany may further compound these stressors, creating a sense of instability and insecurity [16]. Another examination of this refugee group suggests that social isolation and a lack of social support networks may play a significant role in improving the mental health outcomes of female Ukrainian refugees in Germany [17]. Many of the Ukrainian women are separated from their families or communities as a result of their journey to safety.

The complexity surrounding the circumstances of female Ukrainian refugees underscores the imperative need for an in-depth exploration of the factors that influence their mental health outcomes and the coping mechanisms they employ after arrival in Germany. By delving into the subjective narratives of female Ukrainian refugees, the current research aims to uncover the unique stressors, cultural factors, and social dynamics that influence their mental well-being. Furthermore, the results promote a participatory approach, allowing refugees to contribute their perspectives and voices to the discourse on mental health, thereby fostering empowerment and agency within the community.

The current study seeks to achieve the following objectives:

1. To provide a descriptive exploration of the mental health and quality of life outcomes of female Ukrainian refugees
2. To explore the diverse factors influencing the mental health outcomes of female Ukrainian refugees in Germany.
3. To identify the coping mechanisms that female Ukrainian refugees employ to manage the challenges they face after arriving in Germany.
4. To examine the barriers to and facilitators of mental health support and integration services for female Ukrainian refugees in Germany.

## 2. Materials and Methods

### 2.1. Study Design

This study employs a mixed methods approach, combining qualitative and quantitative methodologies to understand the mental health outcomes and resources of female Ukrainian refugees in Germany. The qualitative component facilitates an in-depth exploration of the participants’ lived experiences, challenges, and emotional responses, providing rich contextual insights into the factors influencing their mental health. Meanwhile, the descriptive quantitative method identifies and analyses patterns and trends between mental health and quality of life outcomes. By integrating these two approaches, the study aims to capture the personal and statistical dimensions of refugee well-being, ensuring a holistic perspective on the factors that shape their coping strategies after arrival in Germany. Ethical clearance was obtained from the ethical committee of the Competence Center Health of the Hamburg University of Applied Sciences (approval granted on 21 March 2022).

### 2.2. Sampling and Participants

Participants were recruited using a convenience sampling technique, with recruitment information disseminated through personal contacts and social media platforms (such as Facebook). A purposive sampling strategy was employed to ensure representation from various demographic and socioeconomic backgrounds. Despite the small sample size, our careful selection process aimed to include women from different age groups, education levels, and professional statuses to capture a wide range of perspectives. Fifteen female Ukrainian refugees (aged 18 to 56, M = 38.3 years, SD = 10.3) participated in focus group discussions and completed a quantitative questionnaire. This sample size allows an in-depth examination of the participants’ experience and a comprehensive representation of their subjective perspectives, which facilitates a detailed analysis of collective patterns and avoids burdening the information processing process [18]. Of the participants, two had no formal educational qualifications, four had completed secondary education (second stage), eight held bachelor’s degrees, and one had a master’s degree. Nine participants were married, three were divorced, and the remaining three were single or unmarried. Twelve participants arrived in Germany with one (n = 6) or two children (n = 2). The length of stay in Germany ranged from 7 to 13 months, with an average duration of 10.2 months (SD = 2.4) for all participants.

### 2.3. Qualitative Study—The Focus Group Discussion

Three focus group discussions were conducted in Hamburg in April 2023. These discussions offered a flexible and adaptable platform for participants to engage in subjective evaluations of their mental health, allowing for an in-depth exploration of the factors influencing their well-being and the coping mechanisms they employed [19,20].

The focus group interview was conducted in Ukrainian by a native speaker. The interview guide was initially developed in English and then translated into Ukrainian. A back-translation was performed to ensure the accuracy and consistency of the content. This guide was based on the research objectives and the relevant literature on refugee mental health, ensuring that the key topics were thoroughly addressed. It focused on various themes, including mental health challenges, resources, the impact of displacement, and how participants sought support in their new environment. Open-ended questions were crafted to allow participants to express their views naturally, with follow-up prompts designed to encourage deeper reflection and conversation.

The focus group discussions were held in a comfortable and neutral setting to ensure participants felt safe and at ease. Efforts were made to create an atmosphere of trust and mutual respect where participants could freely share their experiences without fear of judgment. The moderator was trained on flight and (mental) health and spoke Ukrainian fluently. The moderator began by introducing the purpose of the study, emphasizing the confidentiality of the discussions and the importance of respectful listening. Participants were reassured that their perspectives were valuable and that there were no right or wrong answers.

During the focus group discussions, the moderator used active listening techniques, allowing each participant to speak while encouraging quieter members to contribute. This ensured that all voices were heard and that the discussion was inclusive. The discussions were participant-driven, with the moderator remaining flexible, allowing the conversation to evolve naturally. This adaptability enabled the exploration of topics that participants deemed necessary, enriching the data collection process.

Each FGD lasted approximately 90 min, involved an average of five participants, and was audio-recorded with participants’ consent to ensure accuracy in transcription and analysis. After each FGD, participants were debriefed, and their questions or concerns were addressed, reinforcing the collaborative and respectful nature of the process. By integrating a structured interview guide with an open and flexible discussion format, the focus group discussions provided rich qualitative data that complemented the quantitative findings.

### 2.4. Quantitative Approach

In addition to the focus group discussions, a paper–pencil questionnaire was administered to collect data on participants’ demographic characteristics and subjective mental health evaluation. The questionnaire incorporated three scientifically validated tools: the General Health Questionnaire (GHQ-12) [21], the EUROHIS-QOL-8 [22], and the Patient Health Questionnaire (PHQ-4) [23]. These instruments were selected to provide a comprehensive assessment of mental health and quality of life among female Ukrainian refugees in Germany. The questionnaires were available and administered in Ukrainian.

The GHQ-12 is a widely used screening tool designed to detect psychological distress and potential psychiatric disorders. Comprising 12 items, it assesses various symptoms, such as anxiety, depression, social dysfunction, and loss of confidence. Each item is rated on a 4-point Likert scale, capturing how frequently participants experienced these symptoms over the past few weeks. This questionnaire is particularly valuable in identifying non-specific psychological distress, which is common in displaced populations, offering insights into the mental health status of refugees. The GHQ-12 has been validated across diverse cultures, making it highly suitable for refugee populations [21].

Complementing the GHQ-12, the EUROHIS-QOL-8 is a brief version of the World Health Organization’s Quality of Life assessment, designed to measure the overall subjective well-being across crucial life domains. It includes eight items that cover physical and psychological health, social relationships, and environmental factors. Participants rate their quality of life on a Likert scale, responding to questions, like “How would you rate your quality of life?” and “To what extent do you feel your life is meaningful?” This tool is particularly effective in refugee studies due to its adaptability across cultures, allowing researchers to capture refugees’ perceptions of their quality of life in their new environment [22].

The PHQ-4 is an ultra-brief screening tool combining two items from the PHQ-9 (depression) and two from the GAD-7 (anxiety), providing a focused evaluation of the most common mental health concerns in displaced populations. Participants indicate how frequently they have experienced symptoms of anxiety and depression over the past two weeks, on a scale from “not at all” to “nearly every day”. The PHQ-4’s brevity makes it an efficient tool in resource-limited settings while providing crucial insights into the emotional well-being of participants [23].

These three instruments—GHQ-12, EUROHIS-QOL-8, and PHQ-4—offer a holistic view of participants’ mental health and quality of life. The combination allows for a nuanced exploration of quantifiable subjective mental health outcomes and coping mechanisms among female Ukrainian refugees in Germany.

### 2.5. Data Analysis

#### 2.5.1. Qualitative Analysis

The recorded focus group discussions were transcribed using the intelligent verbatim technique, which captures the essence of the participants’ responses while omitting filler words and irrelevant content. To protect their confidentiality, participants were randomly coded. Once transcription was completed, the transcripts were subjected to content analysis to identify patterns, themes, and critical insights related to mental health and resources. Three research team members were involved in the coding process. We employed a two-step approach, where each coder independently coded a subset of the data, followed by collaborative discussions to resolve discrepancies and achieve consensus.

Data analysis was conducted stepwise using a deductive coding technique, as outlined by Kuckartz and Rädiker (2022) [24]. In the initial coding round, the primary focus was on classifying mental health problems and identifying relevant contributing factors. These categories were drawn from preexisting theoretical frameworks and the literature on refugee mental health. Once the mental health issues were classified, further rounds of coding were conducted to examine how participants coped with these issues, paying particular attention to coping strategies and support systems.

In the later stages of analysis, axial and selective coding were applied to refine the emerging themes and focus on specific concepts: short sentences or single words were used to capture the essence of participants’ mental health resources. Axial coding was used to group related subcategories under overarching themes. For example, “Individual Stressors” encompassed subcategories, such as “Acculturation Stress” and “Uncertainty about Returning to Ukraine”. Similarly, other themes, such as “Psychosocial Stressor” and “Structural Stressor”, were identified by examining relationships between categories. Selective coding then integrated these themes into a core narrative that reflected the participants’ experiences. This iterative process ensured that the analysis captured the depth and complexity of the data while aligning with the study’s objectives.

By utilizing these qualitative analysis techniques, the study generated rich, contextual insights into the mental health challenges faced by female Ukrainian refugees and how they navigated these difficulties. The combination of deductive, axial, and selective coding ensured a structured yet flexible approach to data interpretation, facilitating a comprehensive understanding of the participants’ experiences.

#### 2.5.2. Quantitative Analysis

Given the small sample size of 15 participants, the quantitative analysis focused on descriptive statistics to summarize the data and provide an overview of participants’ mental health and well-being to complement findings from the qualitative data. Sum scores were computed for each construct and subconstruct within the PHQ-4, EUROHIS-QoL-8, and GHQ-12 to assess anxiety, depression, quality of life, and other dimensions of psychological distress. These questionnaires are validated among refugee groups and used in similar research settings [7].

The PHQ-4 included two items each for anxiety and depression, rated on a 4-point Likert scale (0 = “not at all” to 3 = “nearly every day”). The total PHQ-4 score, ranging from 0 to 12, represents the overall psychological distress level of each participant, providing a quick assessment of the severity of anxiety and depression symptoms. The sum scores for anxiety and depression were computed separately by adding the responses from the total score, which was determined by adding the scores of each of the four items. Scores are rated as normal (0–2), mild (3–5), moderate (6–8), and severe (9–12). Furthermore, the sum of the two anxiety-related items provided a score ranging from 0 to 6. A total score equal to or greater than 3 suggests anxiety. Similarly, the sum of the two depression-related items provided a score ranging from 0 to 6. A total score equal to or greater than 3 suggests depression.

The EUROHIS-QoL-8, designed to measure the overall quality of life across physical, psychological, social, and environmental domains, consists of eight items, each rated on a 5-point Likert scale (ranging from 1 = “very poor” to 5 = “very good” or “not at all” to “completely”). The total score is calculated by summing the responses to all eight items, resulting in a score ranging from 8 to 40. Higher sum scores on the EUROHIS-QoL-8 indicate better-perceived quality of life. Descriptive statistics, such as the mean, standard deviation, and range, were used to summarize the participants’ quality of life in various domains, providing insights into their overall well-being and satisfaction with their circumstances [22].

The GHQ-12 is a screening tool for identifying psychological distress. It comprises 12 items related to social dysfunction, anxiety and depression, and loss of confidence. Each item is rated on a 4-point scale (0 = “not at all” to 3 = “much more than usual”), with higher scores indicating more significant psychological distress. The construct is further divided into three subscales (social dysfunction—six items, anxiety and depression—four items, loss of confidence—two items). Items covering social dysfunction related to difficulties in social and occupational functioning were summed to create a total social dysfunction score, reflecting the extent of impairment in daily life activities. The subconstruct on anxiety and depression was measured by summing the items related to feelings of anxiety and depression, providing a score that indicates the severity of emotional distress. Loss of confidence focused on self-esteem and self-assurance. Scores were computed by adding responses to items reflecting participants’ confidence and self-worth. Each subconstruct generated a sum score contributing to the overall GHQ-12 score, ranging from 0 to 36. Descriptive statistics were calculated to provide a detailed overview of the psychological well-being of the participants, including the frequency of specific mental health issues, like anxiety, depression, and social dysfunction [25].

The analysis emphasized descriptive statistics—such as means, standard deviations, ranges, and frequency distributions—to describe the participants’ mental health, psychological distress, and quality of life. This approach highlighted general trends and offered insights into the challenges faced by female Ukrainian refugees in Germany rather than attempting to infer broader conclusions or statistical generalizations.

## 3. Results

This section presents both qualitative and quantitative findings regarding the diverse stressors experienced by Ukrainian refugee women in Germany. It then delves into the mental health resources that these women utilize to navigate their challenges. Finally, we outline the barriers that Ukrainian refugee women encounter in accessing healthcare services in Germany.

Additionally, we will examine the barriers to and facilitators of mental health support and integration services tailored explicitly to female Ukrainian refugees in Germany. This analysis aims to provide a comprehensive understanding of their experiences and highlight potential areas for improvement in support systems.

### 3.1. Quantitative Results

As presented in Table 1, descriptive analysis of the PHQ-4 revealed that about five of the participants were in the normal range for depression and anxiety; another five participants showed mild symptoms of both depression and anxiety, three experienced moderate symptoms, and two participants reported severe symptoms. These findings suggest that a significant portion of the sample struggles with mental health issues, with about one-third experiencing moderate to severe symptoms. When examining depression and anxiety separately, four of the participants exhibited depression symptoms. At the same time, close to half reported anxiety symptoms, highlighting that anxiety was more prevalent within the sample than depression.

Regarding quality of life, participants reported scores ranging from 15 to 38 out of a possible range from 8 to 40. The mean score of 25.8 (SD = 6.9) suggests a relatively lower quality of life, as the midpoint of the possible score range is 24. This indicates that, on average, participants reported somewhat lower levels of well-being and life satisfaction, possibly linked to the mental health issues observed in the sample.

For the GHQ-12, which assesses psychological distress, scores ranged from 5 to 22, with a mean score of 12.8 (SD = 5.1), out of a possible score range from 0 to 36. This relatively moderate mean score suggests that the sample experienced some level of psychological distress, though not at the end of the spectrum. Higher scores on the GHQ-12 indicate more significant distress, and the average score reflects that a substantial portion of participants might be struggling with their mental health. When looking at the specific subdomains of the GHQ-12, the social dysfunction subdomain had scores ranging from 2 to 9, with a mean score of 5.2 (SD = 2.4), suggesting that participants faced moderate difficulty in their social interactions and daily functioning. This further emphasizes the challenges in well-being and social adaptation within the sample.

In the anxiety and depression subdomain, the mean score was 4.9 (SD = 2.6), with scores ranging from 0 to 9 out of a possible range from 0 to 12. This result reflects moderate levels of anxiety and depression, reinforcing the findings from the PHQ-4, where anxiety was notably prevalent. Lastly, scores ranged from 0 to 5 in the loss of confidence subdomain, with a mean of 2.9 (SD = 1.4) out of a possible 6. This indicates that participants experienced moderate levels of diminished self-esteem and confidence, which could contribute to both their mental health challenges and their overall quality of life.

### 3.2. Qualitative Results

#### 3.2.1. Predictors of Mental Health Outcomes Among Female Ukrainian Refugees in Germany

The focus group interviews identified several stressors influencing the mental health of Ukrainian refugee women in Germany, categorized into structural, psychosocial, and individual dimensions. Key individual stressors include acculturation stress, uncertainty about returning to Ukraine, and feelings of guilt. Psychosocial stressors include care work and leaving family members behind. Structural stressors include language barriers, poorer career prospects, and a difficult housing situation.

#### 3.2.2. Individual Stressors

##### Acculturation Stress

Five women described feelings of fear, being overwhelmed, and anxiety in everyday situations. These participants described significant acculturation stress, marked by constant fear, anxiety, and feelings of disconnection. They struggled with daily interactions, a sense of belonging, and reconciling life in the host country with their memories of home. Many expressed longing for their former lives and guilt about their struggles, highlighting the emotional burden and isolation experienced during the adaptation process.

One participant expressed constant worry about doing something wrong (P1 (FGD1)):

*“…I always have fears here. I feel anxious all the time. Everyone says, If you scream or behave badly, they will smile at you, but they will call the police…”*

Another participant spoke about feeling disconnected from Ukraine and Germany (P2 (FGD2)):

*“And this dissonance—when you’re here, everyone is drinking coffee, dealing with everyday problems, but you have extraordinary problems. […] It’s like I’m nowhere. I’m not there anymore because I haven’t lived there for a while, and I’m not fully here either. I can’t fully integrate into society.”*

Another participant expressed a longing for her life in Ukraine and felt guilty for complaining (P1 (FGD3)):

*“Everyone is doing well, and I feel ashamed to complain. Back home, I had everything organised. Now, I feel isolated and miss my social circle.”*

##### Uncertainty About Returning to Ukraine

The participants’ uncertainty about returning to Ukraine reflects inner conflicts between opportunities in Germany and their ties to their home country. While some struggle deeply with life changes and yearn to return, others face dilemmas, like leaving behind relationships or adapting to new roles despite having had better jobs in Ukraine. This ambivalence underscores the emotional and practical challenges of staying and returning.

Three women are still undecided about whether to stay in Germany or return to Ukraine. One woman describes the following (P6 (FGD3)):

*“…. It’s very difficult for them to let something new into their lives. And there are many such women who constantly think about how they can return to Ukraine. For some women, it’s very difficult here.”*

Another participant reported that she had a better job in Ukraine but had now integrated into Germany. This resulted in an inner conflict for her. She reports the following (P2 (FGD 3)):

*“There are, of course, moments when I receive a call from (place) and am asked if I want to come back because they want to reopen the medical centre. …And, of course, I sometimes feel desperate. While I’m cleaning toilets here [laughs], I could be leading my old cool life there. […] I realise that I don’t want to return to Ukraine now, even if I could have a good job there.”*

##### Feelings of Guilt

Feelings of guilt were often linked to their perceived inadequacies or inability to contribute to Ukraine from abroad. One woman described a persistent sense of doing everything wrong. At the same time, another expressed guilt about leaving Ukraine, attributing the decision to her child and feeling she could have been more helpful if she had stayed. These feelings highlight the emotional toll of displacement and self-perceived obligations to their homeland (FGD 2):

*“… the feeling of guilt. I had a moment when I was angry with my child. I was very angry with her because she was the reason why I left. Because if I hadn’t left because of her, I could have done something useful [for Ukraine]. And that was a very strong feeling in the first few months.”*

#### 3.2.3. Psychosocial Stressor

Regarding psychosocial stressors, the participants predominantly discuss the challenges associated with care work and express feeling deeply burdened by leaving family members behind in Ukraine.

##### Care Work

Participants described significant challenges related to care work, particularly in managing the emotional needs of their children. One woman expressed the difficulty of caring for her 13-year-old sister, who constantly longed to return to Ukraine despite the ongoing tension in the area. Another participant highlighted the strain of managing her teenage son’s adjustment to life in Germany, feeling overwhelmed by his discontent and her mental health struggles. A third woman shared the constant disruption caused by her daughter’s tantrums, impacting her daily life. These accounts reflect the emotional and practical toll of caregiving in a foreign country, coupled with the tension of leaving family behind in Ukraine and the uncertainty about returning:

P4 (FGD 1): *“It’s very difficult for my sister too. She is very worried because her mother is not there. She is only 13 years old, and she cries all the time; she wants to go home. […]”.*

Another woman also reports feeling overwhelmed with her teenage son and how this significantly affects her mental health (P1 (FGD1)):

*“This issue of adolescence complicates the mental problems I have even more. I mean, the first child is a teenager, and the other is a very small baby, and I don’t know who is harder for me to bear. Probably with the teenager, it’s much harder for me….”*

Leaving family members behind in Ukraine is perceived as very distressing. Furthermore, many women report an internal struggle between the hope of being able to return to Ukraine and embracing the new life in Germany.

##### Leaving Family Members Behind

Participants expressed deep emotional distress from leaving family members behind in Ukraine. One woman shared the burden of her father, who is in the military and unable to leave. At the same time, another described the difficulty of being separated from her elderly mother and brothers, fearing she might be unable to reach them in an emergency. Additionally, many women noted the struggle of trying to integrate into life in Germany while constantly worrying about the safety of their relatives in Ukraine, highlighting the emotional strain of managing migration and separation from loved ones during a time of crisis.

P5 (FGD2):

*“My whole family stayed in Ukraine. My father is in the military, and he’s not allowed to leave. This issue is very difficult for me [voice trembling].”*

Furthermore, many Ukrainian women had to leave their parents behind because they were unable to flee due to their age, as one woman reports (P4 (FGD2)):

*“… I’m now in a situation where I can’t see my loved ones. Especially my mother and brothers. That’s very difficult…”*

#### 3.2.4. Structural Stressors

Regarding structural stressors, the participants mentioned difficulties learning the German language, limited career prospects, and difficult housing situations.

##### Language Barriers

Nine out of the fifteen women interviewed mentioned language barrier challenges, particularly in learning German. One woman shared how the fear of speaking the language prevents her from enjoying everyday interactions, and that she is even avoiding conversations with neighbors. Another expressed frustration with integration courses that group people with varying language skills, leaving beginners struggling to keep up. The inability to understand or communicate in German led to feelings of isolation and uncertainty, with one participant even stating that not speaking the language made her feel invisible in society. These experiences underline the emotional and practical difficulties of adapting to life in Germany without proficiency in the language.

P3 (FGD 2): *“And for me, it’s very difficult to speak another language or even start speaking, even English. To illustrate, It’s even hard for me to talk to my neighbours… We live in a rental apartment that’s like a family house. So, we constantly encounter our German neighbours. For me, it’s a huge challenge to say something to them—I’m afraid. Sometimes I go outside and think, ’I wish they weren’t anywhere so I wouldn’t have to talk to them.’”*

The inability to understand and speak German triggers fears and uncertainty among the women. One participant even reported the following (P1 (FGD 1)):

*“I don’t understand the language; I am nobody here.”*

##### Poorer Career Prospects

Nine participants expressed frustration with the poorer career prospects in Germany compared to Ukraine. Several women reported difficulties finding suitable jobs, with some feeling stuck in low-paying roles or experiencing setbacks in job applications. One participant highlighted the challenges of not having her qualifications recognized, leaving her with only temporary internships. Another woman shared her sense of loss and frustration, contrasting her current job in Germany with her previous, higher-status career in Ukraine. These experiences reflect the emotional toll and barriers to professional advancement that many Ukrainian women face after migration.

P4 (FGD 1):

*“I’ve tried to find a job. I tried at the employment office, at the Welcome Center, everywhere, and the only thing they could offer me was an internship that you can only do for two months….”*

Another participant reported feeling downhearted because her social status had deteriorated in Germany compared to Ukraine (P2 (FGD 3)):

*“…, of course, I’m sometimes desperate. If I’m cleaning toilets here [laughing] and there, I could lead my old cool life.”*

##### Difficult Housing Situations

Several participants described challenging housing situations in Germany. One woman shared the difficulties of living with a German family, highlighting cultural and ideological differences. Another participant noted that some Ukrainian women living in cramped conditions had become isolated, leading to depression. The lack of space and social interaction made these women feel disconnected from their surroundings. Lastly, a woman from Kyiv expressed the psychological strain of living in a rural village, vastly different from her urban life in Ukraine. These stories emphasize the emotional and mental toll of adjusting to new living conditions after migration.

P5 (FGD 2):

*“It was very difficult to live with another family, especially when you have different views on life and a different mentality.”*

P3 (FGD 1):

*“I spoke with women at an organisation, and everyone tells their own story. They live in a hotel, they go downstairs just to smoke, drink tea, discuss a situation that happened in the hotel, you know, these Ukrainian rumours and such. And then they go back to their rooms….”*

### 3.3. The Mental Health Resources Employed by Female Ukrainian Refugees

Several resources were identified for how Ukrainian refugee women deal with their experiences of war and displacement in Germany. Like the stressors, the mental health resources were categorized into individual and psychosocial strategies.

#### 3.3.1. Individual Strategies

Among the individual mental health resources, the refugee women mentioned an active lifestyle, self-realization, repression, and self-care.

##### Active Lifestyle

Six Ukrainian women expressed that engaging in activities and “having something to do” positively affects their mental health. The results highlight that maintaining an active lifestyle positively impacts their mental health. One participant highlighted the benefits of regular exercise, such as jogging, participating in marathons, and staying busy with travel or simple tasks, like cleaning. This helped her avoid negative thoughts and stay focused on the present. Another woman mentioned her friends, who, despite facing difficulties, cope with their challenges by staying active and embracing new experiences. Their resilience and proactive approach to life were key to overcoming adversity.

P2 (FGD 1):

*“Yes, of course. And you know what else helped me? My active lifestyle. I jog, I travel, I have participated in various marathons. ….then I clean windows just to stay occupied. That’s what helps me.”*

##### Self-Realization

For some Ukrainian women, Germany has represented a fresh start and an opportunity for self-realization. One participant shared that she feels more fulfilled in Germany than she did in Ukraine, noting her personal growth and the international connections she has made. Another woman expressed her aspiration to work as a journalist in Germany despite the challenges and her desire to learn German to achieve this goal. This ambition is fueled by her experience at a demonstration for the war anniversary, where she felt inspired to contribute to the Ukrainian community through journalism.

P5 (FGD 3):

*“I can even say that I feel much more realised than I did back in Ukraine. And I’m doing better than I could be now. Because I am realised in many different directions, I have many international acquaintances.”*

P5 (FGD 2):

*“I understand that it is very difficult to become a journalist here. However, when I was at a demonstration for the anniversary of the war in Hamburg and saw how many Ukrainians were there, I realised that I wanted to learn German to work here as a journalist for Ukraine. I really want to realise this plan.”*

##### Repression

For some women, repression is a coping mechanism to deal with the traumatic experiences of war and displacement. One participant explained that the key to managing her emotions is not to think about her experiences. She believes it becomes overwhelming if she allows herself to reflect on the past. Another woman similarly shared that repressing her thoughts was crucial to her survival. She drew parallels to the survivors of concentration camps, highlighting that those who managed to endure were those who focused on maintaining a routine and not allowing themselves to dwell on the bleakness of their situation.

P4 (FGD 1):

*“The main thing is not to think about it! If you don’t think about it, it’s all fine, but if you start thinking about it…”*

P1 (FGD 1):

*“The main thing is not to think about it. I once read the memoirs of people who survived a concentration camp during the Second World War. First, those who thought it would soon be over gave up, then those who thought it would never end, and only those who had a certain routine survived. Just do something, live somehow…”*

##### Self-Care

Self-care plays a crucial role in maintaining mental health according to the women who were interviewed. One participant emphasized the importance of having alone time each day, believing that taking a break for herself, even if only for a few hours, is vital for her well-being. This personal space allows her to recharge and avoid the pressure of constant responsibilities, such as caring for her child. Another woman expressed a more critical view of psychological support, instead trusting in her methods of self-care, particularly physical activities, to manage stress and challenges. She believes that self-reliance is key to coping with difficult situations.

P2 (FGD 2):

*“It is very important to me to be alone from time to time. […]. This means that I can’t just go on spontaneous trips or spend the night somewhere on my own. It’s very important for me to have two or three hours to myself every day. I don’t have to run off straight away to take my child somewhere or pick them up…”*

P2 (FGD 1):

*“…I believe that you have to take care of yourself [physically] to be able to cope with it all. Well, that’s just my tactic, and maybe I’m wrong.”*

#### 3.3.2. Psychosocial Resources

Five psychosocial resources were identified based on the focus group interviews: support from German people, successful integration of children, social volunteering, exchange with other refugees, and the Internet and social media as a source of information and means of contact with family members.

##### Support from German People

Support from German people was a significant positive aspect for many of the women who were interviewed. Six participants highlighted the assistance they received from Germans, particularly in finding accommodation or being welcomed into German homes. One participant expressed gratitude for the help she and her sister received in securing housing, describing it as a stroke of luck. Additionally, two women emphasized how valuable their interactions with Germans were for improving their German language skills. Despite making mistakes, they felt understood, and the supportive environment helped them build confidence in communicating.

P4 (FGD 1):

*“But I have to say that the Germans helped me with all of this. They helped me find a flat and solved all my problems. I can say that my sister and I were lucky with them.”*

P5 (FGD 2):

*“By the way, I’m very grateful to Germans for that; even if you speak with mistakes, they understand everything. Even if I know that I have two verbs together, and that’s wrong.”*

##### Successful Integration of Children

The successful integration of their children in Germany was a source of fulfillment and motivation for four women. One participant shared that, despite the challenges of starting over, knowing that her child is fully integrated and thriving in Germany brings her great happiness. Another woman mentioned that her children’s success in adapting to life in Germany positively impacts her own mental health, as their progress provides her with additional emotional strength and motivation.

P2 (FGD 2):

*“You have to start from scratch, but I know it’s better for my child here. She is fully integrated here. So, completely. That’s great luck for me.”*

Another woman reported that this also affects her mental health (P4 (FGD 3)):

*“I am quite mentally robust. My children motivate me a lot […].”*

##### Social Volunteering

Three participants reported engaging in social volunteering activities in Hamburg. By helping other Ukrainians, the women feel that they are making a valuable contribution to society and experiencing a sense of self-efficacy. One participant shared her involvement in organizing a psychology club for children. At the same time, another described her role as an interpreter, assisting Ukrainians in accessing healthcare and enrolling their children in schools. These volunteer efforts provide the women with a meaningful occupation and a sense of purpose, helping them stay occupied while supporting their community.

P6 (FGD 3):

*“I try to help our Ukrainians because I know (the German) languages; I go as an interpreter to doctors, to different institutions, to different organisations… so this kind of activity keeps me busy.”*

##### Exchange with Other Refugees

Three participants mentioned that they met up with other Ukrainian refugees, and these gatherings helped them feel more comfortable and connected in Germany. One participant shared how the camaraderie with others in her housing unit created a positive environment, and she appreciated the sense of community. Another participant described a club for Russian- and Ukrainian-speaking women in Hamburg, which organizes weekly events. She often participates in these events, finding them helpful for making new connections and coping with stress. The social exchanges within these communities help the women to maintain a sense of belonging and support.

P4 (FGD 1):

*“… there is a so-called club of Russian and Ukrainian-speaking women who organise various weekly events. I often participate in them and make contacts …, and that helps.”*

##### Internet and Social Media

Two participants mentioned that they use the Internet and social media to gain information on psychological topics, primarily related to coping with trauma and integration. One participant shared how she listened to podcasts by psychologists to understand her feelings better and realize that her struggles were normal. Another participant uses social media to stay connected with her family in Ukraine, posting photos and videos to support the army and raise awareness about the situation in Ukraine. She believes that her social media posts contribute to the cause and help others understand the ongoing conflict.

P5 (FGD 2):

*“…. I started listening to various podcasts by psychologists who discuss this topic. It’s normal not to feel guilty. I realised that there was nothing wrong with me.”*

P5 (FGD 2):

*“I post a lot of photos and videos from Ukraine to support the army and the people because I have a lot of foreign friends on Instagram …. This is my contribution to victory.”*

### 3.4. Barriers and Facilitators to Mental Health Support of Ukrainian Refugee Women

Five women reported challenges in accessing the German healthcare system. One participant mentioned struggling to find psychological support in Hamburg, feeling very depressed, but only finding a few psychological sessions in Lübeck. Another woman, despite being a psychologist herself, found it difficult to secure therapy for her daughter, as many organizations only provide services for adults. Language barriers also created obstacles, as one participant shared her frustration with being sent back and forth for two weeks before finally receiving assistance. Even then, she was required to bring an interpreter to access the necessary treatment, which resulted in additional costs and inadequate communication.

P2 (FGD2):

*“I have the impression that there is a lack of psychological help because I was looking for help. I felt very depressed several times, and all I found were a few psychological sessions in Lübeck.”*

P6 (FGD 3):

*“…I have contacted some organisations that could help me find a psychologist for (her daughter), but many of them only work with adults and not with children.” Language barriers also make access to health care more difficult.*

## 4. Discussion

The key findings of this study are that nearly 43% of participants exhibited anxiety symptoms, and 21% showed signs of depression, indicating high levels of mental health challenges.

Acculturation stress and language barriers significantly contributed to psychological distress among female Ukrainian refugees in Germany.

Active lifestyles, volunteering, and support from German people were identified as primary coping mechanisms for managing stress.

Barriers to accessing mental health services, particularly for children, remain a significant challenge for these refugees.

The current study set out to explore the mental health outcomes and resources employed by female Ukrainian refugees in Germany, utilizing a mixed methods approach that combined both qualitative and quantitative data. By integrating focus group discussions with standardized mental health assessments, the study aimed to provide a comprehensive understanding of the diverse stressors these women face and how they navigate the challenges of displacement and integration. The results suggest that acculturation stress, social isolation, and language barriers are significant contributors to psychological distress, with many participants exhibiting symptoms of anxiety and depression. The focus on coping strategies, such as active lifestyles, community support, and volunteering, adds a significant dimension to the current literature, which often centers primarily on the challenges without adequately addressing the strengths and resources of the refugee population. Generally, these results emphasize how war and individual exposure to war-related events may significantly influence the severity of psychological symptoms, highlighting the need for timely and context-specific interventions to support their well-being.

### 4.1. Objective 1: Descriptive Exploration of the Mental Health and Quality of Life Outcomes

The quantitative analysis presented concerning levels of depression and anxiety among the participants, revealing that 43% exhibited symptoms of anxiety and 21% showed signs of depression. These results align with findings from Buchcik et al. (2023) [7], which documented the widespread prevalence of anxiety and depression among Ukrainian refugees. The elevated rates of anxiety, in particular, can be understood in the context of the traumatic experiences these individuals have faced, including displacement from their homes, loss of loved ones, and ongoing fears related to the war in Ukraine. The average quality of life score of 25.8 (SD = 6.9) suggests that this group experiences lower life satisfaction and well-being than the general population. This is particularly concerning, as low quality of life scores are often indicative of the challenges faced by refugees in adjusting to a new environment while coping with past traumas. The stark contrast in quality of life highlights the need for targeted mental health support to address the unique challenges faced by this population. The GHQ-12 scores indicated moderate psychological distress, further supporting the notion that despite being in a safer environment, the uncertainty of their situation continues to weigh heavily on the refugees. Many refugees grapple with existential questions regarding their future, including fears of instability and the possibility of returning to an unsafe homeland. This uncertainty can exacerbate feelings of anxiety and depression, reinforcing the findings of broader research showing that refugees often experience heightened psychological distress post-resettlement.

Moreover, integration challenges, such as navigating a new culture, establishing social networks, and finding stable employment, can contribute significantly to psychological distress [26,27]. These factors create a compound effect, whereby the realities of adaptation and integration overshadow the initial relief of reaching a safer environment.

While the demographic data presented in this study are comprehensive, the inclusion of education as a variable is particularly relevant when considering both stressors and resources for refugee women. As 9 out of 15 participants received higher education in their home country, this level of education may influence the perception of stressors and the development of coping mechanisms. Higher educational attainment has been shown to correlate with better mental health outcomes, as it can enhance an individual’s ability to cope with stress, increase access to social and economic resources, and improve psychological adjustment [28]. A low level of education has been found to have a significant negative impact on the development of depression and mental health management strategies, particularly in women [29,30] making it an important factor in understanding the overall well-being of Ukrainian refugee women in Germany. Therefore, despite the elevated levels of anxiety and depression found in this study, education may influence coping mechanisms and psychological adjustment.

### 4.2. Objective 2: Explore the Diverse Factors Influencing the Mental Health Outcomes of Female Ukrainian Refugees After Arrival in Germany

The study revealed multiple factors influencing the mental health outcomes of female Ukrainian refugees, categorized into structural, psychosocial, and individual dimensions. These stressors included language barriers, uncertainty about returning to Ukraine, care work, and social isolation.

The prominence of language barriers is consistent with the findings of [31], a scoping review, which indicates that such barriers can lead to significant feelings of isolation among refugees. Without adequate German language skills, these Ukrainian refugees may struggle to engage in social or professional activities, exacerbating their perception of disconnection from the host society. This phenomenon is particularly relevant in Germany, where effective communication is essential for integration and accessing support services [32,33]. Acculturation stress emerged as a dominant factor, with refugees feeling neither fully integrated into German society nor connected to their homeland. This “in-between” state reflects the complex emotional landscape of displacement, where individuals grapple with losing their previous identity while facing challenges adapting to a new culture. Smeekes et al. (2017) highlight this sentiment, emphasizing the emotional toll of feeling uprooted and the challenges associated with forging new identities in a foreign context [34].

Furthermore, psychosocial factors, such as care work, and the absence of a social network, significantly contributed to the stress experienced by participants. The burden of care responsibilities was particularly pronounced among siblings and women separated from their partners, which aligns with the findings of Liu et al. (2020). These women often juggle multiple roles without the support of their partners, which can exacerbate feelings of stress and being overwhelmed. The absence of a robust support network not only limits their emotional resilience but also heightens their vulnerability to mental health challenges [35].

### 4.3. Objective 3: Identify Mental Health Resources Among Female Ukrainian Refugees

The study identified various mental health resources employed by female Ukrainian refugees, categorized into individual and psychosocial strategies. Many participants adopted active lifestyles to maintain mental health, echoing findings from Ekblad et al. (2024), which highlights physical activity as a protective factor in managing stress among refugee populations [36]. Regular exercise serves as a physical outlet and contributes to improved mood and reduced anxiety, fostering resilience in the face of adversity. In addition to physical activity, self-care practices, social volunteering, and support from German residents emerged as significant mental health resources that helped participants navigate their challenges. Volunteering enabled these women to feel a sense of purpose and belonging within their new communities, crucial for countering feelings of isolation and disconnection. This sense of contribution can also enhance their self-esteem and foster social networks, which are essential for emotional support during times of crisis. Furthermore, receiving support from local Germans was vital in reducing feelings of alienation, facilitating smoother integration and providing a sense of community amid their struggles.

However, a subset of participants reported utilizing repression as a mental health resource, deliberately avoiding thoughts of the traumatic experiences they had endured. While this strategy may temporarily relieve distressing emotions, it is essential to recognize the potential adverse long-term effects. As noted by Vromans et al. (2021), unresolved trauma can exacerbate mental health conditions over time, leading to heightened psychological distress and complications in the healing process. Relying on repression can hinder individuals from processing their experiences and seeking appropriate support, ultimately impeding their recovery [37].

### 4.4. Objective 4: Barriers and Facilitators to Mental Health Support and Integration Services

The qualitative findings identified significant barriers to mental health support for female Ukrainian refugees, highlighting challenges, such as difficulties navigating the German healthcare system and the lack of specialized psychological services tailored for refugees. One participant noted the unavailability of psychological help for children, underscoring a critical service gap. This finding parallels the work of Im et al. (2021) [38], who emphasizes that mental health services in host countries are often ill-equipped to address the unique needs of refugees. The complexity of the healthcare system, combined with language barriers and bureaucratic hurdles, can further exacerbate feelings of frustration and helplessness among women who are seeking support.

However, mental health support facilitators included the crucial backing of German residents and the successful integration of children into local schools and communities. Many participants expressed that receiving assistance and kindness from locals provided emotional relief, fostering a sense of belonging and community. This social support mitigated feelings of isolation and encouraged women to seek help more actively.

Albers et al. (2021) have also documented the positive impact of children’s integration, emphasizing that family well-being is often central to refugee women’s mental health. When children adapt well to their new environment—forming friendships and engaging in educational activities—it can alleviate stress for mothers, allowing them to focus on their mental health needs. The successful integration of children serves as a vital facilitator, contributing to the overall emotional stability of families and promoting resilience among mothers [39].

### 4.5. Study Strengths and Limitations

One of the strengths of this study is the use of a mixed methods approach, allowing for a more holistic understanding of the participants’ experiences. Integrating qualitative and quantitative data provided statistical insights into mental health outcomes and a deep exploration of the personal factors contributing to these outcomes.

However, concerning the quantitative part, the study is limited by its small sample size, which limits the generalizability of the findings. The recruitment method—convenience sampling—may have also led to a selection bias, as participants who were more socially connected or better informed about the study were more likely to participate. This is reflected in the high number of participants with higher degrees, which may not be representative of all Ukrainian refugee women in Germany, potentially limiting the broader applicability of the results. Furthermore, the study only included female participants, which limits the exploration of mental health outcomes across other demographic groups within the refugee population.

As for the educational background, 9 out of the 15 participants hold a higher degree (bachelor’s or master’s), which may not fully reflect the broader population of Ukrainian refugees, it is important to note that individuals with higher education may be more likely to engage in online platforms and research opportunities. This discrepancy highlights the need for caution when generalizing the findings and points to the potential for future studies with more diverse samples to better capture the experiences of refugees from varying educational and socioeconomic backgrounds.

## 5. Conclusions

This study provides valuable insights into the mental health outcomes of female Ukrainian refugees in Germany, demonstrating that acculturation stress, social isolation, and language barriers are significant stressors affecting their well-being. Despite these challenges, refugees employ various coping mechanisms, such as maintaining active lifestyles, volunteering, and receiving support from local Germans. However, access to mental health support remains a critical barrier, underscoring the need for more inclusive healthcare services.

The findings highlight the necessity for more targeted mental health interventions designed explicitly for female Ukrainian refugees. Given the prominence of language barriers as a stressor, integrating language support services into mental health care is essential to ensure that refugees can access the necessary support effectively. Moreover, mental health services must be tailored to the unique needs of refugee women, particularly in addressing care responsibilities and the psychosocial impacts of leaving family members behind.

Policymakers should consider offering more comprehensive support systems that encompass psychological services, career development opportunities, and social integration programs. By doing so, we can create an environment that addresses immediate mental health needs and fosters long-term well-being. Future research could explore the long-term impact of these interventions and how they may alleviate psychological distress over time. By bridging the gaps identified in this study, we can enhance the quality of life and mental health outcomes for this vulnerable population, fostering better integration and resilience in their new environment.

## Figures and Tables

**Table 1:** Descriptive results on participants’ mental health outcomes after arrival in Germany (n = 15).

	M	SD
Quality of life	9.4	8.2
GHQ Aggregate Psychological Distress	12.8	5.1
GHQ Social Dysfunction	5.2	2.4
GHQ Anxiety and Depression	4.9	2.6
GHQ Loss of Confidence	2.9	1.4
	**N**	**%**
PHQ-4		
Aggregate Score (Depression and Anxiety)		
Normal	5	33.3
Mild	5	33.3
Moderate	3	20
Severe	2	13.3
Anxiety		
No anxiety symptoms	8	53.3
Anxiety symptoms	7	46.7
Depression		
No depression symptoms	11	73.3
Depression symptoms	4	26.7

Notes: GHQ = General Health Questionnaire [21]. Quality of life measured by the EUROHIS-QOL-8 [22], PHQ–4 = Patient Health Questionnaire (PHQ-4) [23].

## Data Availability

The data presented in this study are available on request from the corresponding author.
